# All-Direction Random Routing for Source-Location Privacy Protecting against Parasitic Sensor Networks

**DOI:** 10.3390/s17030614

**Published:** 2017-03-17

**Authors:** Na Wang, Jiwen Zeng

**Affiliations:** School of Mathematical Science, Xiamen University, Xiamen 361005, China; wangna@stu.xmu.edu.cn

**Keywords:** wireless sensor networks, random routing, source-location privacy protecting, identity authentication

## Abstract

Wireless sensor networks are deployed to monitor the surrounding physical environments and they also act as the physical environments of parasitic sensor networks, whose purpose is analyzing the contextual privacy and obtaining valuable information from the original wireless sensor networks. Recently, contextual privacy issues associated with wireless communication in open spaces have not been thoroughly addressed and one of the most important challenges is protecting the source locations of the valuable packages. In this paper, we design an all-direction random routing algorithm (ARR) for source-location protecting against parasitic sensor networks. For each package, the routing process of ARR is divided into three stages, i.e., selecting a proper agent node, delivering the package to the agent node from the source node, and sending it to the final destination from the agent node. In ARR, the agent nodes are randomly chosen in all directions by the source nodes using only local decisions, rather than knowing the whole topology of the networks. ARR can control the distributions of the routing paths in a very flexible way and it can guarantee that the routing paths with the same source and destination are totally different from each other. Therefore, it is extremely difficult for the parasitic sensor nodes to trace the packages back to the source nodes. Simulation results illustrate that ARR perfectly confuses the parasitic nodes and obviously outperforms traditional routing-based schemes in protecting source-location privacy, with a marginal increase in the communication overhead and energy consumption. In addition, ARR also requires much less energy than the cloud-based source-location privacy protection schemes.

## 1. Introduction

**Figure 1:**
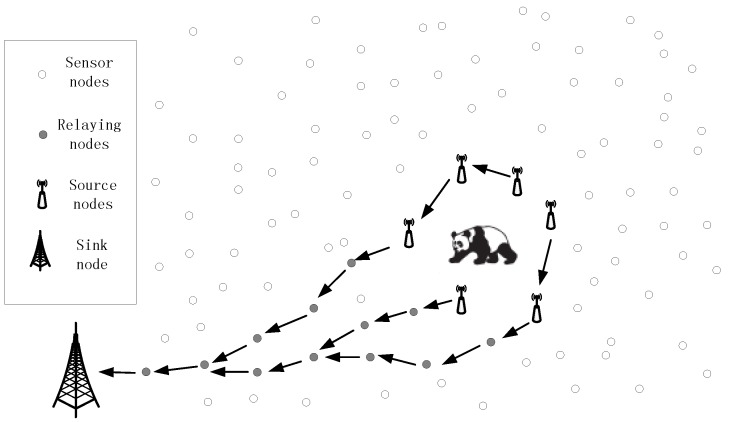
A scenario of target monitoring.

Supported by the rapid development of information techniques, circuit engineering, sensor technology, and artificial intelligence, wireless sensor networks (WSNs) have been widely used in the fields of habitat monitoring, target tracking, and military surveillance [1,2,3,4,5]. As an example, a WSN used to monitor pandas’ wild habitat [6] is presented in Figure 1. When detecting a panda, the sensor nodes around the panda are always kept active and collect valuable information of the panda in a collaborative way. Then, a stream of packages is delivered to the sink node via a proper routing algorithm. In general, for each routing algorithm in the WSN, there is an objective function in terms of timeliness, energy saving, robustness, or package delivery success, and so on. As an example, the shortest path routing algorithm always tries to find the shortest path from the source node to the sink node, in order to transmit the packages; the directed diffusion routing algorithm always selects the path with the highest value for timeliness to deliver the packages; to deliver the packages in a totally distributed way and improve the robustness of the routing process, the GPSR routing algorithm always employs the Greedy Forwarding Pattern to move the package in the networks and employs the Perimeter Forwarding Pattern to recover the package, when Greedy Forwarding Pattern fails. In conclusion, for all of the routing algorithms with a constant objective function in WSNs, the selection of the next hop always obeys a constant rule and, as a result, the packages always tend to select a set of similar routing paths if the packages have similar sources and destinations. In Figure 1, the shortest path routing algorithm is employed to select the next hop of the packages. We can observe that, in the process of delivering the packages generated by the nodes around the panda, all of the routing paths are strongly related to each other. Further, in the process of delivering the packages from the source nodes to the sink node, the routing paths are closer and closer to each other and, eventually, all of the paths will converge to a single path.

The inherent characteristics of traditional routing algorithms discussed previously make it possible for the adversaries to trace the packages back to the source nodes and find the pandas. If we always employ a constant routing algorithm in a WSN to deliver the packages from the source nodes to the sink nodes, the routing paths will always be similar to each other and the strong adversaries can easily trace them back to the source nodes, based on the parasitic sensor networks. Therefore, it is unnecessary for the adversaries to deploy a very large network with a big investment and they only need to deploy some parasitic nodes in the original network to collect the contextual privacy information which can be used to locate the pandas. Via proper strategies, it is easy for the adversaries to find the pandas in a short amount of time, with a very small cost. This is extremely unfair for the owners of the network, who use a lot of effort to construct the network, and may further threaten the safety of the valuable asset monitored by the network. As a result, it is urgent for us to solve the source location privacy problem.

**Figure 2:**
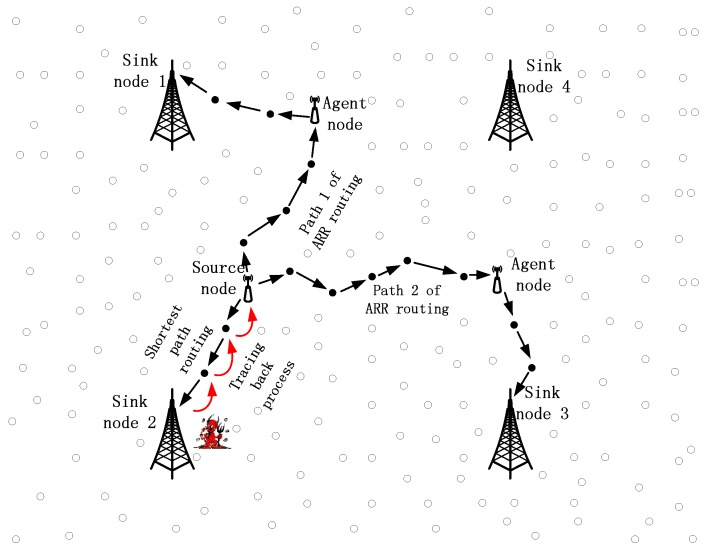
Shortest path routing and ARR routing.

Consider an extremely large network in which a large number of sensor nodes are redundantly scattered and a number of sink nodes are deployed. When a node detects a specific target, it sends the packages to any one of the sink nodes, i.e., it is possible for a package to be sending them in any direction, rather than to a constant sink node. Figure 2 presents a part of the whole network with four sink nodes and the source node locations in the region controlled by these four sink nodes. In most of the traditional routing algorithms, the source node will choose a sink node with the shortest distance and send the packages to that sink node. Taking shortest path routing as an example, the source node always sends packages to sink node 2 if a target stays around this source node for quite a while. As a result, the parasitic nodes can easily track the package back to the source node, guided by the red arrows, as shown in Figure 2. Besides, we can produce similar conclusions for some other rouging algorithms, such as the GPSR and directed diffusion algorithm.

To protect source location privacy, several routing-based approaches have been proposed, which assume that the parasitic nodes have a limited overhearing capability, e.g., similar to a sensor node’s transmission range. This is reasonable considering that the adversaries can’t monitor the whole network at the same time; otherwise they wouldn’t need to employ a parasitic network. Further, the parasitic nodes are initially deployed around the sink nodes and trace back the hop-by-hop movement of the packages to find the locations of the source nodes. Routing-based schemes preserve the source nodes’ location privacy by delivering the packages through different paths, instead of a set of similar paths, to improve the difficulty of tracing back the packages.

In this paper, we design a novel all-direction random routing algorithm (ARR) for source-location protecting against parasitic sensor networks. ARR can select the routing paths in a very flexible way, which can guarantee that the routing paths are totally dispersive and an accidental observation of a part of a routing path is useless for the parasitic nodes. ARR is composed of three modules, i.e., selecting a proper sink node and an agent node; delivering the packages to the agent node from the source node; delivering the packages to the sink node from the agent node. The source node first selects a sink node with a probability that has a reverse relationship with the distance between the source node and the sink nodes. Then, an agent needs to be selected in an indirect way, because the agent node may be located far from the source node and the source node doesn’t know the topology of the whole network. In fact, the source node just needs to decide a “location” around the sink node and the node nearest to the “location” is the agent node. Then, the packages with the “location” destination are sent by the source node and the packages are delivered to the “location”, based on the geographic routing algorithm. Further, we designed a perimeter-based approach to help the packages be accurately transmitted to the agent node, even if the source node doesn’t know the exact agent node. Once the agent node receives the packages, it can employ any routing algorithm and sends the packages to the sink node.

For a same source node, it can choose many different routing paths to deliver the packages to the sink nodes. What is more, the source node can control the rough shapes of the paths, based on only local decisions, and as a result, the paths can be very different for the same source node. As shown in Figure 2, path 1 and path 2 of ARR are totally different and it is impossible for parasitic nodes around sink nodes to trace the packages back to the source, based on the random paths.

The main contributions of this paper can be summarized as follows: (1) we propose a new scheme to construct shared keys between neighboring nodes, which is integrated with an identity authentication function to defend against the joining of parasitic nodes; (2) we propose a novel all-direction random routing approach which can significantly improve the diversities of the routing paths, with a very marginal increase in the communication overhead; (3) we conduct a series of experiments to evaluate the performance of the proposed routing algorithm with that of shortest path routing, greedy perimeter stateless routing, and phantom routing.

The rest of the paper is organized as follows: we first review the routing-based source-location privacy protecting approaches in Section 2. Network and parasitic node models are presented in Section 3, in which the attack process is also presented. To defend the attack, we design a random routing algorithm in Section 4 and its performance is evaluated in Section 5. At last, we conclude this paper and present our future research plan in Section 6.

## 2. Related Work

In recent years, source-location privacy in wireless sensor networks has gained much attention and many approaches have been proposed in the literature. Based on the parasitic nodes’ models, the approaches can be roughly divided into two categories, i.e., global-adversary-based schemes and local-adversary-based schemes.

In global-adversary-based schemes, the adversaries can monitor all the traffic of the entire network and all the collected information can be used to analyze the source locations of the packages [7]. In this case, the best choice to defend against adversaries is sending dummy packages to confuse the adversaries and most of the existing approaches attempt to identify a good balance between the security of the source node, the overhead of dummy packages, the package delivery delay, and the quality of service. Shao et al. [8] proposed a statistically strong source location privacy protecting scheme to decrease the time delay of pakages, without significantly increasing dummy packages, in which the source node sends real packages as soon as possible, while keeping them statistically indistinguishable from the dummy packages. Lu et al. [9] designed a location privacy scheme for cluster-based WSNs, in which the cluster heads can filter the dummy packages to decrease the overhead of dummy packages. Then, the cluster heads periodically send the real packages to the sink node, resulting in a long time delay. Similar to [9], the dummy packages are also filtered in the network by a proxy node to decrease package transmission [10], and for different proxy assign patterns, the lifetime of the networks are discussed.

Most local-adversary-based source location protecting schemes try to design novel routing algorithms to make it more difficult to track the routing paths. The phantom routing technique proposed in [11] is composed of two phases, i.e., a random walking phase and a subsequent flooding/single path routing phase. In order to avoid the steps of random walking canceling each other, two directed random walk techniques are designed, including a sector-based directed random walk and a hop-based directed random walk. After the random walk phase, the sensor nodes can employ any existing routing algorithm to deliver the packages to the final destinations. However, a phantom routing algorithm cannot control the accuracy of the location where the first phase turns to the second phase, and as a result, some paths may be similar to each other, which can decrease the safety of the source location. Further, the parameter k, which is preset by the users to control the walking hops of a package in a random walking phase, is very difficult to set considering that the densities of the nodes for different regions are very different. For densely deployed nodes, the destination of k steps of a random walk is near to the source and cannot protect the source location very well; for sparsely deployed nodes, a relatively smaller k is acceptable because the distances between the nodes are large and a large k will increase the package transmission of the whole network. Wang et al. [12] formulate the source-location privacy protecting problem as an optimization problem in terms of the average or minimal trace back time for the adversaries to reach the source node from the sink node. Then, a random parallel routing and a suboptimal but practical privacy-aware routing algorithm named the weighted random stride are proposed, to protect the source-location. In [13], the packages are modified and routed by dynamically selected nodes to make it difficult for the adversaries to trace the packages back to the source nodes. In the scheme, a rehash seed is used to determine the intermediate nodes, to reconstruct the packages and then send the packages to the sink node.

In addition to the routing-based schemes, some other schemes are also proposed in the literature. Recently, a cloud-based scheme [14] for protecting source-location privacy is proposed, in which an irregular shaped cloud filled with fake packages is constructed around the source node. Though the adversaries can track the packages back to the boundary of the cloud, it is very difficult to find the accurate location of the source node.

## 3. Network and Parasitic Node Models

We first assume an extremely large 2-D WSN composed of a large amount of homogeneous sensor nodes, which is used to track targets and is monitored by some parasitic nodes. Each node can locate itself in a proper manner and knows the locations of the sink nodes, which are always the destinations of the packages. Obviously, each node can easily learn of its neighbors’ locations, based on one communication behavior. To decrease information transmission and save energy, we further assume that the network contains multiple sink nodes, rather than only one sink node. This is reasonable considering that the routing paths are too long if only one sink node exists in the network, no matter where it is located. To deploy these sink nodes, we first divide the network into squares of the same size and then the sink nodes are deployed manually on the vertices of the squares, as shown in Figure 2. These sink nodes are logically equivalent and the source node can send the packages to any one of the sink nodes. Then, each pair of sink nodes can communicate with each other directly and share information through wireless channels or wired links, which are pre-installed by the users. The deployed network employs the k-nearest neighbors tracking approach proposed in [15], to track different types of targets. Each node follows a sleeping schedule and keeps silent when no target is detected. However, if a node detects a target in its duty regions, it needs to remain active until the target moves out of its duty regions. Once a target is detected by some nodes, these corresponding nodes immediately and accurately locate the target in a cooperative manner and send the information of the target to any one of the sink nodes.

We assume that the adversaries try to locate the source nodes of the packages based on the contextual information of the WSNs [16]. As an example scenario in [11,14], the hunters want to track the source nodes and then further find the pandas, which are of great value. We assume that the adversaries deploy several complicated parasitic nodes with supporting equipment such as spectrum analyzers and communication models, to obtain the information of traffic distribution. At the beginning, the parasitic nodes are uniformly deployed around the sink nodes, considering that the locations of targets are uniformly distributed in the whole network and all the destinations of the packages are the sink nodes. If a parasitic node observes that a package is sent from node $n_{i}$, it moves to node $n_{i}$ and waits until it hears another package being sent from node $n_{j}$. Then, the parasitic node moves to node $n_{j}$ and repeats the process until it reaches the place near to the source node. Further, we assume that the parasitic nodes can communicate with each other and make decisions in a collaborative way.

## 4. All-Direction Random Routing Algorithm

### 4.1. Pre-Deployment Phase

**Figure 3:**
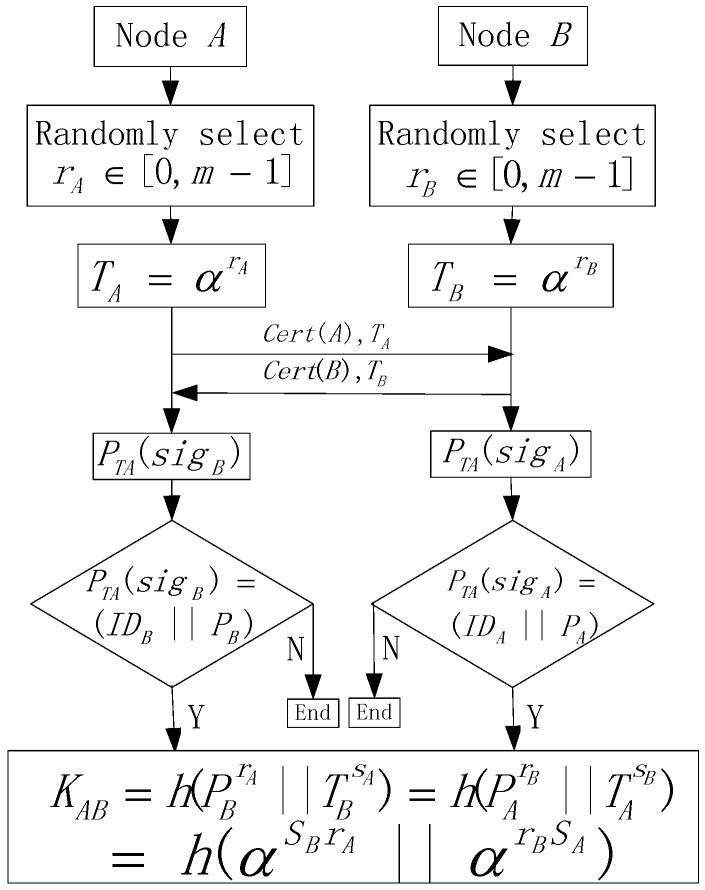
Flowchart of constructing shared keys by node a and b.

Before deploying the network, each sensor node $n_{i}$ needs to be loaded with a unique identifier $ID_{n_{i}}$, public key $P_{n_{i}}$, and secret key $S_{n_{i}}$, to construct secure communication links with other nodes. The network operator first generates a cyclic multiplicative group (G,) and an element α∈G with an order m. Then the secret key $S_{n_{i}}$ of node $n_{i}$ is selected from [0,m−1], and the corresponding public key is computed by $P_{n_{i}}=\alpha^{S_{n_{i}}}$. This is reasonable according to Discrete Logarithm Difficulty [17,18], given α and $P_{n_{i}}$, and no algorithm can compute $S_{n_{i}}$ in the expected polynomial time. Further, to defend against the joining of parasitic nodes, each node in the network must have the ability to verify the legality of other nodes. Therefore, we further assume that there is a trusted authority controlled by the operator of the network, denoted by TA with a secret key $S_{TA}$, and a trusted verification key denoted by $P_{TA}$. It is worth noting that the trust authority is not deployed in the network and its safety is guaranteed by the operators of the network. Then, each sensor node $n_{i}$ in the network is loaded with a certificate $Cert(n_{i})=(ID_{n_{i}}||P_{n_{i}}||sig_{n_{i}})$, where $ID_{n_{i}}$ is the unique identifier of node $n_{i}$, $P_{n_{i}}$ is node ${n_{i}}^{\prime }s$ public key, and $sig_{n_{i}}=S_{TA}\left(ID_{n_{i}}||P_{n_{i}}\right)$ is the signature of the TA. We present the flowchart used for verifying the legality of each node and further constructing the shared key by node A and B, in Figure 3.

First, node A and B randomly select two numbers in the range of [0,m−1], i.e., $r_{A}$ and $r_{B}$, respectively, and they compute $T_{A}=\alpha^{r_{A}}$ and $T_{B}=\alpha^{r_{B}}$, based on $r_{A}$ and $r_{B}$. Suppose that node A wants to verify the legal identity of node B, node A needs to request the certificate from node B and then it can verify the legality of node B by checking whether $P_{TA}\left(ID_{B}||P_{B},sig_{B}\right)=true$. In the same way, node B can check the legality of node A. If at least one node of A and B is a parasitic node, the process ends. However, if both of A and B are legal nodes of the network, they can further generate the shared key $K_{AB}$. Node A can compute $K_{AB}=h(P_{B}^{r_{A}}||T_{B}^{S_{A}})$ and node B can compute $K_{BA}=h(P_{A}^{r_{B}}||T_{A}^{S_{B}})$. Considering that $P_{A}=\alpha^{S_{A}}$, $T_{A}=\alpha^{r_{A}}$, $P_{B}=\alpha^{S_{B}}$ and $T_{B}=\alpha^{r_{B}}$, we can ascertain that $K_{AB}=h(\alpha^{S_{B}r_{A}}||\alpha^{r_{B}S_{A}})=K_{BA}$ and the two nodes are able to successfully construct the shared key.

Compared with traditional pairwise key construction approaches, we integrate a legality verify function, which has been widely researched [19], into the shared key construction process, which is used to identify the parasitic nodes and exclude them from the network. This is of great value considering that some important information about the source nodes may be intercepted by the parasitic nodes once they are pretending to be legal nodes in the network. In the proposed process, the mainly employed modular exponent operations are relative time- and energy-efficiency compared and, as a result, are suitable for WSNs.

### 4.2. Selection of the Sink Node and the Virtual Location L Which Defines the Agent Node

As presented in Section 3, we assume that each node knows its own location and all the locations of the sink nodes which are destinations of the packages. Each four neighboring sink nodes define a square with these four sink nodes as vertices, and if a source node locates in the square, the destination of its packages can be only one of these four sink nodes. If the adversaries deploy the same number of parasitic nodes around each sink node, the best strategy for the source node is to send the packages to the four sink nodes with an equal probability. On the other hand, if the source nodes send packages to the nearest sink node with a higher probability, the average energy consumption decreases. In conclusion, there is a tradeoff between source-location privacy security and energy-efficiency. Assume that the distances between the source node and these four sink nodes $s_{1},s_{2},s_{3},\;\mathrm{and}\;s_{4}$ are $d_{1},d_{2},d_{3}$, and $d_{4}$, then the probability of sending the package from the source node to the sink node $s_{i},i=1,2,3\;or\;4$ is defined as follows:

(1) $$P_{s_{i}}=\alpha \times \frac{1}{4}+\left(1-\alpha \right)\times \left(1-\frac{d_{i}}{d_{1}+d_{2}+d_{3}+d_{4}}\right),i=1,2,3\;or\;4.$$

When α is equal to one, the four sink nodes have equal probabilities of being the destination of the package and it is the very difficult for the adversaries to track the package back; with the decreasing of α, the distances between the source node and the sink nodes increase and perform more important roles in the probabilities; and when α is equals to zero, the probabilities are totally dependent on the distances and it is more energy-efficient. In this paper, we attempt to effectively protect the location privacy and we set equal probabilities for these four sink nodes.

Another challenge is selecting the agent node. Obviously, the agent node can’t be too close to the source node or to the sink node. In such cases, the shapes of the routing paths would not change much, compared with traditional routing approaches. In this paper, we employ a two-dimensional normal distribution $N\left(M_{1},M_{2},V_{1},V_{2},\rho \right)$ to randomly generate the virtual location L, which defines the agent node in an undirected way. The agent node is the node in the network that is nearest to L. The parameters of the normal distribution significantly affect the shape of the routing paths and we discuss how to set the parameters in the following.

**Figure 4:**
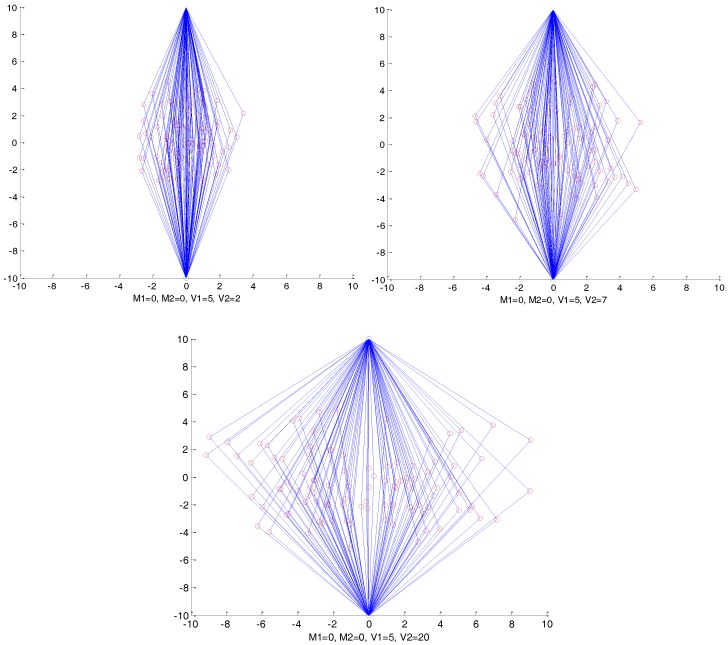
Distributions of agent nodes with different parameters.

We first assume that the location of the source node is $\left(x_{1},y_{1}\right)$ and the sink node’s location is $\left(x_{2},y_{2}\right)$ and that ρ is set to zero, which has a very limited affection on the shape of the routing paths. Then, both $M_{1}$ and $M_{2}$ are set to $\left(\frac{x_{1}+x_{2}}{2},\frac{y_{1}+y_{2}}{2}\right)$, which is the center of the two-dimensional normal distribution. The variances of $V_{1}$ and $V_{2}$ significantly affect the distribution of the routing paths. Consider Figure 4 as an example, where $M_{1},M_{2}$, and $V_{1}$ are set to 0, 0, and 5, respectively, and we set $V_{2}$ as 2, 7, and 20. We can observe that, with the increasing of $V_{2}$, the routing paths become more and more distributed in crosswise patterns, which makes it much harder to track the packages back. Similarly, $V_{1}$ controls the vertical distribution of the routing paths.

**Figure 5:**
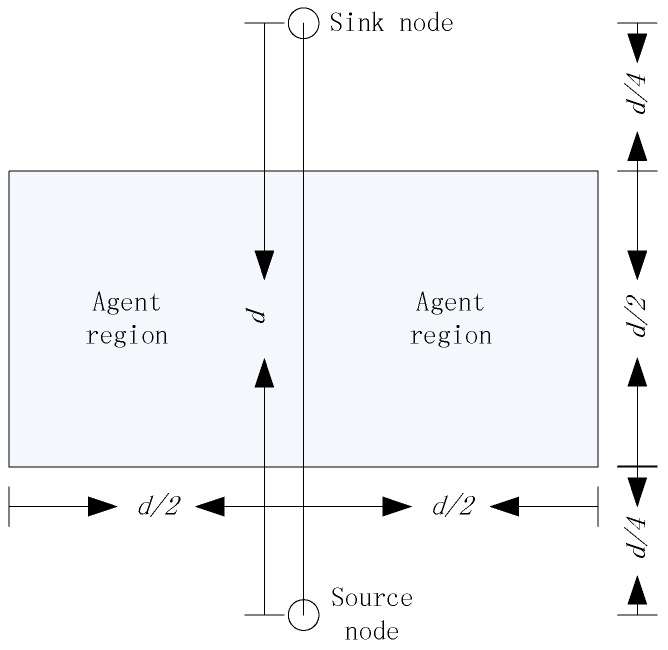
Agent region employed in this paper.

Intuitively, it is necessary to control the region of the agent nodes. As an example, we don’t want to select the agent nodes which are too close to the source node and sink node, because the routing paths would be very similar to existing routing algorithms. Further, the agent nodes can’t be too far from the source node and sink node, which would increase the energy consumption of delivering the packages. When we set $V_{1}={\left(d/12\right)}^{2}$ and $V_{2}={\left(d/6\right)}^{2}$, where d is the distance between the source node and the sink node, we can infer that the target would locate in the agent region shown in Figure 5, with a probability higher than 99% based on the Three Sigma Rule.

In our opinion, the agent region in Figure 5 is large enough to hide the routing paths. Even if the parasitic nodes can trace the packages back to the agent node, there is no value for the adversaries to trace this back to the source node. However, in theory, the agent node can be anywhere in the network and it is flexible for the users of the network to set the rules of selecting the agent nodes, according to the security requirements. Obviously, with the increasing of $V_{1}$ and $V_{2}$, both the size of the agent regions and the security of the source nodes increase, and as a result, the average hops of the package delivery increases, which also increases the energy consumption of the networks. Considering that the agent nodes can’t be too close to either the source node or the sink node, in this paper, we always set $V_{1}={\left(d/12\right)}^{2}$ and $V_{2}={\left(d/6\right)}^{2}$, without special declaration.

In conclusion, having detected a target, each source node randomly selects a sink node as the destination of the packages and then calculates the distance to the sink node as d, as shown in Figure 5. Based on the locations of source node, sink node, and d, the source node can easily obtain all the parameters which are used to generate L. Note that, the whole process presented in this subsection can only be operated by the local computing of the source nodes. Obviously, L is a location rather than a real sensor node and the source node needs not know whether there is a sensor node accurately locating at L. As discussed previously, the agent node is defined as the nearest sensor node to L and, in this way, there is always an agent node. In the next section, we present how to deliver the packages to the agent node, defined by L in a distributed way.

### 4.3. Package Delivery from Source Node to Agent Node Defined by L

As discussed in the previous section, the source node selects the agent node in an indirect way. It first provides a virtual location and the agent node is defined as the nearest node to the virtual location L in the whole network. In this section, we present the process of package delivery from the source node to the agent node. In the initial stage of this process, the source node generates a package of $\left(M,S_{i},L,D\right),$ where M is the mode of the package including two types, i.e., delivering the package from the source node to the agent node and from the agent node to the sink node. $S_{i}$ is the final destination of the package and it must be one of the sink nodes, L is the virtual location which is used to find the agent node, and D is the valuable data on the monitored targets which needs to be sent to the sink nodes. Then, the source node sends the package to one of its neighbors.

For each node n receiving a package, it first checks the mode M of the package. If the package is sent from the agent node to the sink node, the processing steps are discussed in Section 4.4. If the package is sent from the source node to the agent node, node n first employs the Encroach Forwarding Pattern to select the next hop of this package. Using the Encroach Forwarding Pattern, node n first scans all its neighbors and finds all of the legal neighbors with a shorter distance to location L, compared with the distance between n and L. In GPSR [20], the next hop is the neighbor with the shortest distance to the destination. Considering that a constant rule for selecting the next hop is beneficial for the adversaries to analyze the routing paths, node n randomly selects a neighbor in the set of legal neighbors in the Encroach Forwarding Pattern.

The Encroach Forwarding Pattern may fail at a node x when a local optimal result is found, and then. we need to employ the Perimeter Forwarding Pattern to recover it. However, the packages can turn back from Perimeter Forwarding to Encroach Forwarding, if a closer node to L is found compared with x. A planar graph structure has an important role in the perimeter forwarding pattern and we first briefly introduce two planar graphs in the following. The Gabriel graph (GG) [21] and relative neighborhood graph (RNG) [22] are two long-known planar graphs and can divide a plane into several non-overlapping polygons. Both of these two structures can be constructed in a distributed way, as shown in [20]. In this paper, we employ the GG structure, in which each pair of nodes can only transmit packages to each other under the Perimeter Forwarding Pattern when the link between both nodes is a legal edge [22].

**Figure 6:**
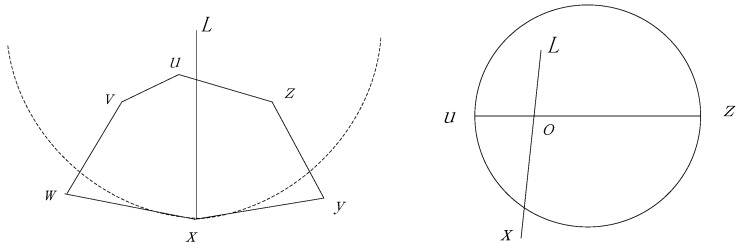
The destination locates out of the polygon and its simplified form.

The Perimeter Forwarding Pattern is operated on the planar graph and employs the right-hand rule to traverse the corresponding polygon which is intersected by the line xL if x is the node in which the package turns to the Perimeter Forwarding Pattern. As shown in Figure 6, a package with destination L turns to the perimeter pattern at node x, because none of the neighbors has a smaller distance to L. If the destination of L is out of the polygon, the distances between u or z to the destination L must be smaller than that of x to the destination L. Therefore, the perimeter forwarding can always change to the Encroach Forwarding Pattern at node u or z when L is out of the polygon, and we prove this in Theorem 1.

**Theorem** **1.** *In the Perimeter Forwarding Pattern, if the destination of the package* L *locates out of the polygon intersected by the line* xL*, where* x *is the node that the package turns to the Perimeter Forwarding Pattern, the package can always turn to the Encroach Forwarding Pattern at one of the nodes on the polygon*.

**Proof.**  As shown in the left section of Figure 6, we assume that a package turn from an Encroach Forwarding Pattern to Perimeter Forwarding Pattern at node x and L, locates out of polygon $P_{uvwxyz}$, which is intersected by the line xL. We further assume that line xL intersects with line uz at point o, as shown in the right section of Figure 6. Without the loss of generality, we assume that o is closer to u, compared with z. Considering that line uz is a legal edge in the GG graph, we can infer that x must locate out of the circle, with uz as the diameter. Then, we can observe that xL=xo+oL>uo+oL>uL. As a result, the perimeter pattern can turn to the Encroach Forwarding Pattern at node u. On the other hand, if o is closer to z compared with u, the perimeter pattern can turn to the Encroach Forwarding Pattern at node z. In conclusion, the package can always turn to the Encroach Forwarding Pattern at one of the nodes in the polygon. ☐

Another possible situation is that a package turns to the Perimeter Forwarding Pattern at node x and L locates in a polygon $P_{x\cdot }$ which contains x as one of its vertices. In this condition, we prove that the agent node defined by L is one of the vertices of polygon $P_{x\cdot }$ in Theorem 2.

**Theorem** **2.** *If a package turns to the Perimeter Forwarding Pattern at node* x *and* L *locates in a polygon* $P_{x\cdot }$ *which contains* x *as one of its vertices, the agent node defined by* L *is one of the vertices of polygon* $P_{x}$*.*

**Figure 7:**
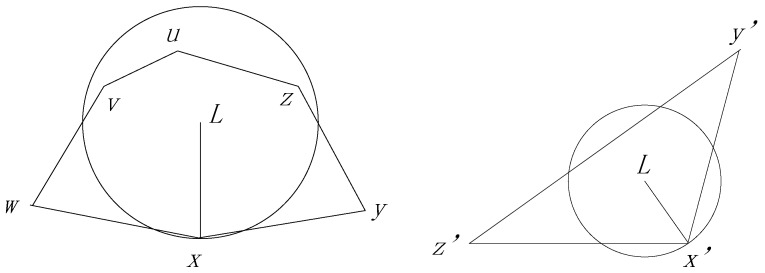
The destination locates out of the polygon and a simplified form.

**Proof.**  If L locates in the polygon, as shown in the left section of Figure 7, we need to prove that one of the nodes $n^{\prime }$ on the polygon has the smallest distance to L among all the sensor nodes in the network. Then, $n^{\prime }$ is the agent node decided by L. We first assume a simplified case presented in the right section of Figure 7. We extend point L as a circle until it touches a node $x^{\prime }$ on the polygon $P_{x}$. If the circle directly touches the node without touching any other edge of the polygon, $x^{\prime }$ is the closest node to L. In some cases, the circle touches a few edges before it touches $x^{\prime }$, as shown in the following Figure. The areas of the circle which are outside of the polygon must be covered by the circles, with the edges as diameters. Based on the properties of the GG graph, we can infer that no other sensor nodes have a smaller distance to L compared with $x^{\prime }$ and, as a result, we know that $x^{\prime }$ is the agent node. ☐

**Figure 8:**
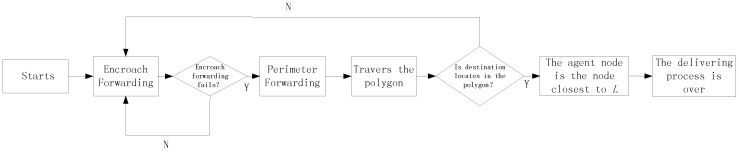
The flowchart of delivering a package from the source node to the agent node.

In conclusion, the packages are always transmitted by the Encroach Forwarding Pattern if possible and only employ the Perimeter Forwarding Pattern when the Encroach Forwarding Pattern fails. Further, the Perimeter Forwarding Pattern turns back to the Encroach Forwarding Pattern or the agent node if it is found successfully. Based on the previous discussion, the flow chart of delivering a package from the source node to the agent node is presented in Figure 8, which guarantees that we can always find the agent node based on L in a distributed way.

### 4.4. Package Delivery from Agent Node to Sink Node

Once the agent node receives a package from the source node, it first changes the mode M of the package, to deliver the package from the agent node to the sink node. In this process, any existing routing algorithm for WSNs can be employed to deliver the package. However, considering that both GPSR and the proposed approach in this paper are geographic-based routing algorithms and they need very similar geographic information, the best choice is choosing GPSR as the routing algorithm of this process. In this case, the construction of the planar graph can be fully used and the whole random routing approach can be fully operated in a distributed way. Note that, a stream of packages coming from the same source node may be delivered to different sink nodes and each sink node cannot observe the whole picture of the monitored target. Therefore, once the package is received by one of the sink nodes, the node needs to share the package with its neighbors to construct the whole picture, which is meaningful to the users.

### 4.5. Analysis and Discussion of ARR

**Figure 9:**
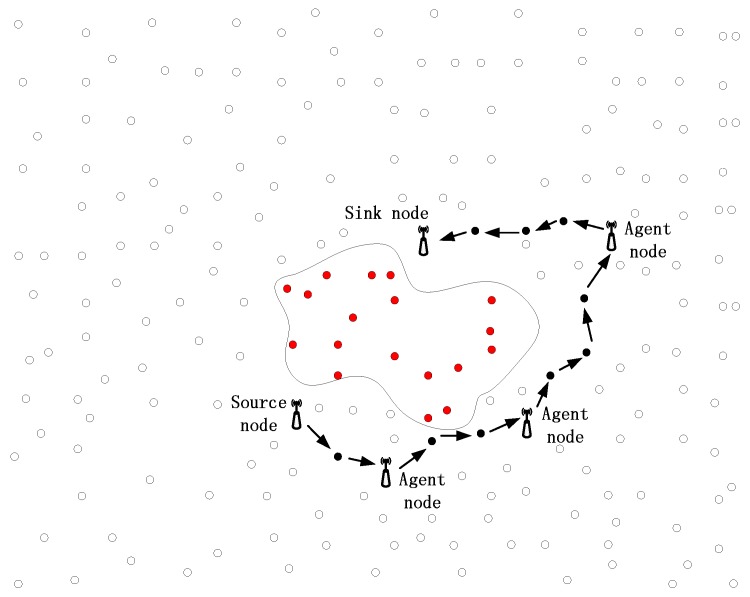
Updated ARR applied in WSNs with dangerous regions.

In this paper, only one agent node is selected in the process of delivering a package from the source node to the sink node. Intuitively, ARR can be easily updated and used in some other applications through different methods of choosing numbers of agent nodes when delivering a package. Consider the application scenario shown in Figure 9. We assume that the red nodes are compromised by the adversaries and the regions occupied by these nodes are called dangerous regions. When delivering packages, the dangerous regions should be avoided, considering that the compromised nodes may extract valuable information from the packages. Because the dangerous regions have high dynamics, most of the existing routing algorithms can’t solve this problem very well, without a significant increase in the energy consumption.

However, we can slightly modify ARR to easily solve the problem. Knowing the accurate geographic locations of the whole dangerous region, the source node can roughly select an agent node away from the dangerous region that has a closer distance to the sink node. Note that, the distance between a node and the sink node is not the Euclidean Distance, considering that the packages need to be delivered around the dangerous region and the distance is the hops of successfully delivering the package to the sink node, without crossing the dangerous region. Then, each agent node chooses the next agent node in the same way, until the packages can be directly transmitted to the sink node with a very low possibility of crossing the dangerous region. With a proper number of agent nodes, the routing paths can tightly cling to the dangerous region and it is the optimal routing algorithm to solve the dangerous region problem.

## 5. Performance Analysis and Evaluation

In this section, we use NS-3 discrete events simulator to evaluate ARR in terms of source nodes’ location privacy preservation, average time delay of delivering a package from the source node to the sink node and average energy consumption in Section 5.1, Section 5.2 and Section 5.3, respectively. As discussed in Section 3, the whole monitoring region is divided into squared regions and each region is controlled by four sink nodes locating at the four corners of the region. As a result, the whole network is composed of many similar squared regions and for the sake of convenience, we conduct our experiments on just one squared region of the whole network. This is reasonable considering that the simulation results of the whole network are the sum of that of all the squared regions and all the squared regions have similar simulation results, in theory. Therefore, in our experiments, we uniformly randomly scatter 10,000 sensor nodes in a 4000 m×4000 m squared field and deploy four sink nodes at the four corners of the squared region. The parasitic nodes are initially deployed around the sink nodes and they try to track back to the source nodes, step by step. The monitoring target is randomly generated in each simulation and it employs a random walk model with a speed of 1 m/s. In the simulation, the WSN employs the k-nearest neighbors tracking approach [15] to monitor the targets and sends the collected information to sink nodes once a target is monitored.

The simulation parameters are summarized in Table 1 as follows.

We compare the performance of the proposed approach with that of shortest path routing algorithm, phantom routing algorithm, and the cloud-based scheme. The random routing hops of the phantom routing algorithm are set to 20% of the hops between the source node and the sink node. The number of nodes contained in the cloud for the cloud-based scheme is set to six times the number of the hops between the source node and the sink node. Therefore, with the increase of the distance between the source node and the sink node, the cloud also increases, and this is intuitive. We end the simulation if the distance between a parasitic node and the source node is smaller than 50 m or 10,000 packages, i.e., the monitoring process lasts for 10,000 s, are successfully delivered to the sink nodes. Each simulation is operated 100 times and the average simulation results are presented.

### 5.1. Source Node’s Location Privacy Protection

**Figure 10:**
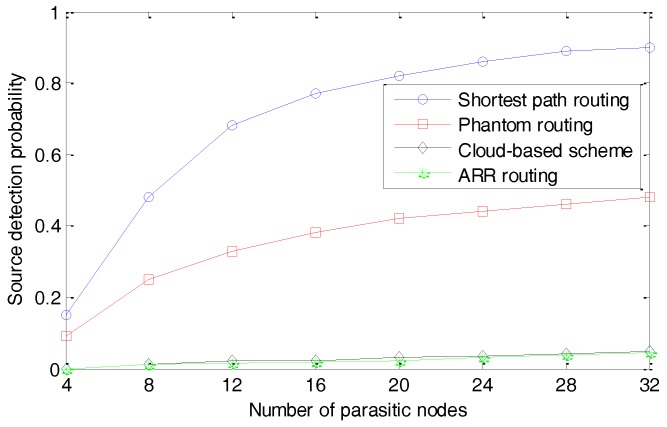
Source detection probability with different numbers of parasitic nodes.

**Figure 11:**
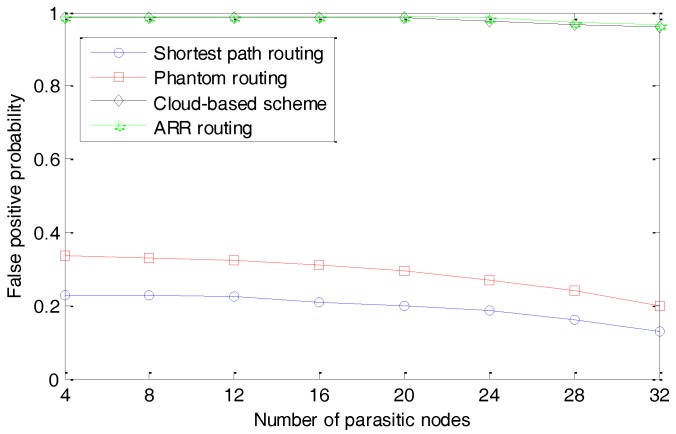
Source detection false positive probability with different numbers of parasitic nodes.

In this section, two metrics called the source detection probability and the false positive probability are employed, to evaluate the performance of the proposed approach in terms of privacy preservation. The source detection probability is defined as the probability that the parasitic nodes can locate the source nodes successfully. In our simulation, it is measured by the number of times that the parasitic nodes locate the source nodes to the total number of simulation runs. The false positive probability is defined as the probability that the parasitic nodes falsely identified a node as the source node, and it is measured by the number of times that the parasitic nodes identified a node as a source node to the total number of times that the parasitic nodes suspected that a node was a source node. The decrease of source detection probability and increase of false positive probability indicate a stronger protection of the schemes. With a different number of parasitic nodes, the source detection probabilities and false positive probability are presented in Figure 10 and Figure 11, respectively.

We can observe that, with the increasing number of parasitic nodes, the source detection probabilities increase for all the four schemes. The shortest path routing algorithm can’t provide protection to the source-location privacy, considering that it always chooses similar routing paths for the same source node and sink node. As a result, it is very easy for the adversaries to trace back to the source node and when the adversaries deploy 32 parasitic nodes, they can find the source node with a probability higher than 90%. The phantom random routing algorithm significantly outperforms the shortest path routing algorithm, which can be explained by the fact that a random walking process is employed to confuse the adversaries. However, Phantom can’t provide a strong enough protection of the source-location, because the adversaries can find the source nodes with a probability of 40% when they deploy more than 16 parasitic nodes. Both the cloud-based scheme and ARR provide very strong protection to the source-location privacy. In the cloud-based scheme, the parasitic nodes can be close to the boundaries of the cloud, but they can’t locate the source node accurately, considering a large amount of fake packages. ARR performs well, because its routing paths are very different to each other, even for the same source node and sink node. Considering that the parasitic nodes can move forward one step when they detect a package and the next path of another package would be very far away, the trace back process would be interrupted and the parasitic nodes can’t get any information from a single package. As a result, the adversaries can’t find the source nodes, though they locate the source nodes several times in a random way.

As shown in Figure 11, with the increasing number of parasitic nodes, the false positive probabilities decrease for all the four schemes, indicating that the adversaries can locate the source node with an increasing accuracy. This is reasonable considering that the monitored information of all the parasitic nodes can be fully used by the adversaries. However, the cloud-based scheme and ARR routing perform much better than the other two schemes. In conclusion, ARR and the cloud-based scheme demonstrate a similar performance in terms of protecting the source-location privacy and they perform much better than the other two schemes.

### 5.2. Average Time Delay with Different Hops

**Figure 12:**
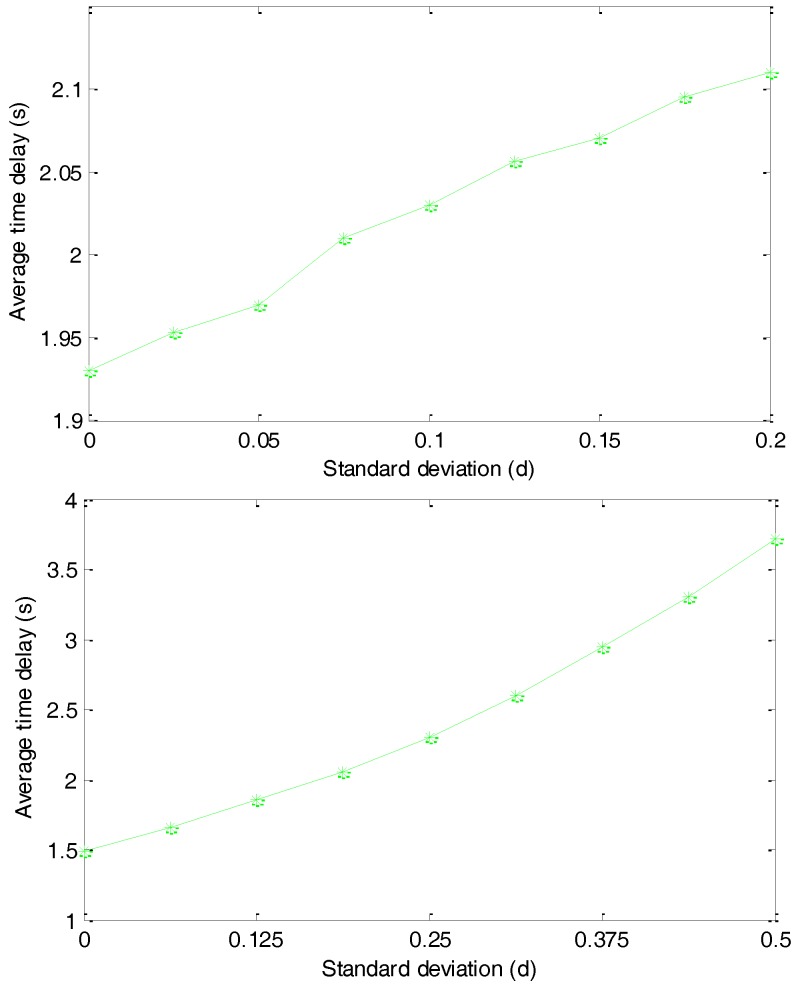
Average time delay with different standard deviation $\sqrt{V_{1}}$ and $\sqrt{V_{2}}$.

As discussed in Section 4.2, the size of the agent region has a direct influence on the average lengths of the routing paths and we present the simulation results in Figure 12. We first set $V_{2}={\left(d/6\right)}^{2}$ and change the standard deviation $\sqrt{V_{1}}$ from 0 to 0.2d. As shown in the top of Figure 12, with the increasing of $\sqrt{V_{1}}$, the average time delay increases very slowly and the average time delay increases by about 15% when we increase $\sqrt{V_{1}}$ from 0 to 0.2d. This result indicates that, although $V_{1}$ can significantly affect the diversity of the routing paths, it has a very limited affection on the lengths of the routing paths.

We then set $V_{1}={\left(d/12\right)}^{2}$ and change $\sqrt{V_{2}}$ from 0 to 0.5d. The simulation results presented in the bottom of Figure 12 illustrate that with the increasing of $\sqrt{V_{2}}$, the average time delay of delivering a package from the source node to the sink node monotonously and significantly increases. The average time delay increases by about 150% when we increase $\sqrt{V_{2}}$ from 0 to 0.5d. This is reasonable considering that $V_{2}$ has a much larger affection on the average lengths of the routing paths. In the following simulations, we assume $V_{1}={\left(d/12\right)}^{2}$ and $V_{2}={\left(d/6\right)}^{2}$.

**Figure 13:**
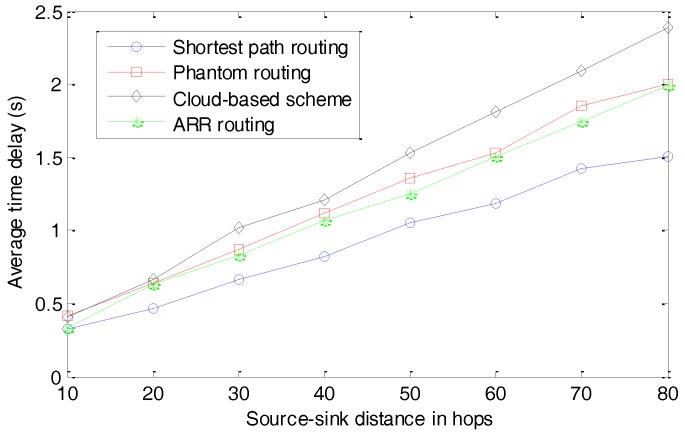
Average time delay with different hops.

The average time delays for different schemes are presented in Figure 13. The source-sink distance in hops is defined as the number of hops when delivering a package from the source node to the sink node through the shortest path routing algorithm. We can observe that the shortest path routing algorithm can always deliver the packages to the sink node with the smallest time delay. This is reasonable because of its objective function when designing the routing algorithm. The Phantom routing algorithm and ARR exhibit similar performances in terms of the average time delay. Both of them slightly extend the lengths of the routing paths to confuse the parasitic nodes. Fortunately, because these two routing algorithms can be operated in a distributed manner and perform well in dynamic networks, they perform better than the cloud-based scheme. In the cloud-based scheme, considering that the clouds need to be updated periodically and the locations of the fake source nodes can’t be controlled by the source node, the average time delay is the largest out of all the four schemes.

### 5.3. Energy Consumption

**Figure 14:**
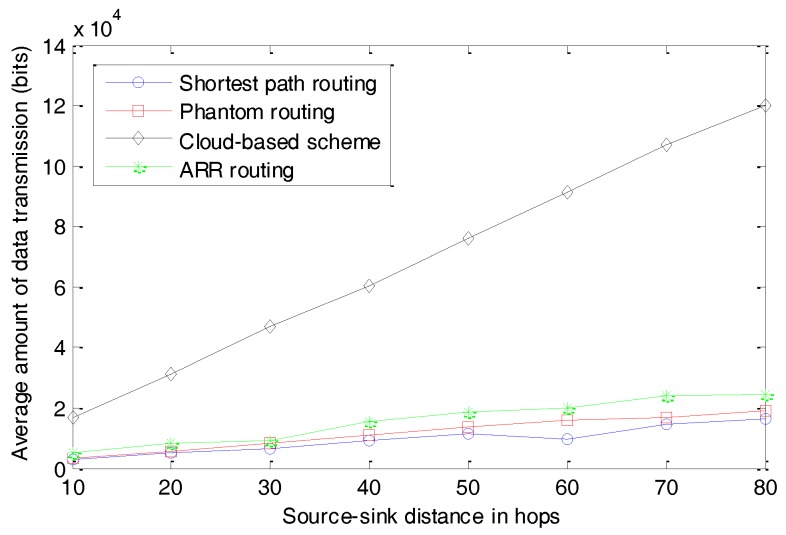
Average amount of data transmission per round.

**Figure 15:**
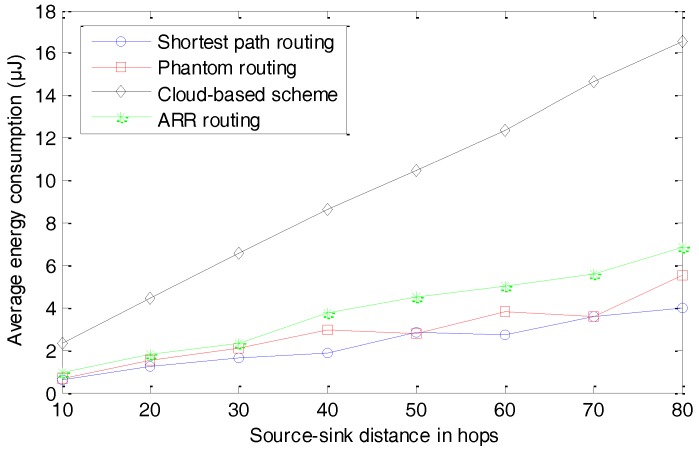
Average energy consumption per round.

Another very important concern in WSNs is energy consumption, which has a strong relation with the amount of data transmission and the complexities of algorithms executed by the sensor nodes. We first present the average amount of data transmission per round, as seen in Figure 14. In our simulation, a round is defined as the whole process of monitoring a target, generating a package, and successfully delivering the package to the sink node. All the data transmitted in the whole network are taken into consideration. Further, we present the average energy consumption per round in Figure 15.

As shown in Figure 14, the three routing-based schemes, including shortest path routing, Phantom routing, and ARR routing, transmit much less data than the cloud-based scheme. In the cloud-based scheme, many fake packages are transmitted in the cloud to make the real package indistinguishable. In most cases, the number of fake packages is much larger than that of the real packages, and as a result, most of the energy is consumed by the fake packages. In our simulation, the average amount of energy consumed in the cloud-based scheme is about five to seven times that of the other three schemes. Though the three routing-based schemes exhibit similar performances, ARR and the Phantom routing algorithm transmit slightly more data compared with the shortest routing algorithm. This can be explained by the fact that the packages have a slightly longer path in ARR and the Phantom routing algorithm.

The simulation results of average energy consumption are presented in Figure 15. With the increase in the distance between the source node and the sink node, the average energy consumption increases. Similar to the data transmission, the cloud-based scheme consumes much more energy than the other three schemes, because of the fake packages. ARR and the Phantom routing algorithm consume slightly more energy than the shortest routing algorithm, because of their longer routing paths.

In conclusion, ARR and the cloud-based scheme can provide much stronger source-location privacy protection than the shortest routing and Phantom routing algorithms. However, the time delay of the cloud-based scheme is the largest of all the four schemes. In addition, the other three routing-based schemes are much more energy-efficient compared with the cloud-based scheme. ARR performs very well in all of the three metrics including source-location protection, the time delay of packages, and energy-efficiency.

## 6. Conclusions and Future Work

In this paper, we proposed an all-direction random routing algorithm to defend against parasitic sensor networks which are employed to trace packages back to the source nodes. Our scheme can be operated in a distributed way and each node needs to make local decisions. The source nodes have the highest authority to control the routing paths of packages, considering that they know all the historical routing paths and can make the best choices. A source node controls the shape of a routing path by first selecting a virtual location which further defines the agent node in an indirect way, i.e., the sensor node nearest to the virtual location in the whole network. Then, a complicated mechanism is designed to deliver the packages from the source node to the agent node, and finally, the packages are delivered from the agent node to the sink node. It is extremely difficult for the parasitic nodes to trace this activity back to the source nodes, because the agent nodes are carefully selected and they are very dispersive. Therefore, with the same source node and sink node, the routing paths are totally different with each other. Simulation results illustrate that the proposed approach can provide very strong source-location protection, with an acceptable increase in the total package transmission of the whole network.

In our future research, we will attempt to design a two-fold source location privacy protecting scheme in which an anonymity cloud is first constructed around the source nodes to hide the source nodes and a distributed random routing algorithm is then designed, based on the geographic information, to send the packages from the fake source nodes to the sink nodes. This approach will provide all-around protection to source-location privacy in WSNs.

## Figures and Tables

**Table 1:** Simulation parameters.

Parameter	Value
Size of the network	4000 m × 4000 m
Number of nodes	10,000
Number of sinks	16
Number of targets	1
$R_{c}$	30 m
Adversaries’ hearing range	30 m
Number of parasitic nodes	$N_{p}$
$V_{1}$	${\left(d/12\right)}^{2}$
Target monitoring scheme	k-nearest neighbors tracking
Event transmission rate	1 s
Length of data in package S	1024 bit
Length of the head of a package	32 bit
